# Cross cultural adaptation, reliability and validity of the Persian version of the university of Wisconsin running injury and recovery index

**DOI:** 10.1186/s12891-024-07171-0

**Published:** 2024-01-09

**Authors:** Bahram Sheikhi, Hadi Akbari, Bryan Heiderscheit

**Affiliations:** 1 Department of Biomechanics and Sport Injuries, Kharazmi University, Tehran, Iran; 2 Department of Sport Sciences, University of Zabol, Zabol, Iran; 3 Department of Orthopedics and Rehabilitation, Department of Biomedical Engineering, University of Wisconsin-Madison, Madison, WI US

**Keywords:** Cross cultural adaptation, Validity, Reliability, Running related injury, Persian

## Abstract

**Background:**

The University of Wisconsin Running Injury and Recovery Index (UWRI) was developed to evaluate running ability after a running-related injury. The aim of this study was to translate and cross-culturally adapt the UWRI into Persian (UWRI-Persian) and to investigate its psychometric properties in patients with a running-related injury.

**Methods:**

The UWRI-Persian was translated using the Beaton guidelines. One hundred and seventy-three native Persian patients with running-related injuries were participated in the study. The exploratory factor analysis was carried out using the principal component analysis method with Varimax rotation. The construct validity of the UWRI-Persian was evaluated using the Pearson correlation with the pain self-efficacy questionnaire (PSEQ), Tampa scale for Kinesiophobia (TKS), and visual analogue scale (VAS). Test-retest reliability was tested among 64 patients who completed the form again after seven days.

**Results:**

The UWRI-Persian showed excellent internal consistency for total score (α = 0.966). An excellent internal consistency (α = 0.922) was shown for psychological response and good internal consistency (α = 0.887) for running progression. The interclass correlation coefficient for the UWRI-Persian total scores was 0.965 (95% CI, 0.942 to 0.979), indicating high intra-rater reliability. The UWRI-Persian showed a moderate correlation with the PSEQ (*r* = 0.425) and the TSK (*r* = 0.457) and a weak correlation with the VAS (*r* = 0.187). These findings suggest no floor or ceiling effects.

**Conclusions:**

The UWRI is a reliable and valid tool for Persian-speaking patients with running-related injuries. The UWRI was successfully translated from English to Persian and demonstrated good to excellent internal consistency, validity and reliability with no floor or ceiling effects.

**Supplementary Information:**

The online version contains supplementary material available at 10.1186/s12891-024-07171-0.

## Background

Running is one of the most common activities that gives rise to acute/overuse injuries to the lumbar spine and lower extremities [1, 2]. Regardless of the type of injury, running-related injuries (RRI) diminish pleasure in training and are associated with undesirable consequences, including temporary or permanent discontinuation of running and work absences. Despite the fact that some programmes have been implemented to reduce the risk of injury, rehabilitation [3, 4], and return to running [5], the management of RRI remains a major challenge.

A thorough understanding of the most common RRIs is an essential step in developing effective injury prevention, rehabilitation and return to running training that can reduce the high incidence or prevalence of RRIs. Several objective and functional outcome measures are available to assess recovery after RRI [6–8].

Monitoring RRI recovery using valid measurements that incorporate sport-specific features is paramount because each sport involves unique physical and psychological demands. For this reason, questionnaires/indices or scales should be specific to the sport discipline and to the construct being assessed. The University of Wisconsin Running Injury and Recovery Index (UWRI) is a novel, running-specific, patient-reported outcome measure that reflects how runners assess their running ability while recovering from an RRI [9].

The UWRI is a simple, self-administered measurement tool and responds to changes in running function after an RRI. The original English version of the UWRI has been previously cross-culturally adapted to Spanish, German and Turkish [10–12]; but it has not yet been translated into Persian. Prior research indicates that an analysis of psychological and risk factors influencing the prevalence of RRI in Iranian runners reveals that 54% of the runners have reported experiencing at least one RRI [13, 14]. Considering that there are several million Persian speakers worldwide who also show great interest in running, adapting the UWRI into Persian may assist researchers to design appropriate rehabilitation and return to running programmes. Therefore, the aim of this study was to translate, and cross-culturally adapt the UWRI into Persian and to investigate the main psychometric properties of the UWRI-Persian.

## Materials and methods

This cross-sectional study was assessed and approved by the Ethics Committee of the University of Zabol (Approval ID: IR.UOZ. REC.1402.001) prior to data collection. The study was performed in accordance with the ethical standards in the World Medical Association Declaration of Helsinki [15]. It was performed in two parts; part 1 involved the adaptation process of UWRI into standard Persian language. Part 2 focused on the psychometric properties analysis of the adapted inventory. The Consensus-Based Standards for the Selection of Health Measurement Instruments (COSMIN) include various measurement properties, such as content validity, construct validity, internal consistency, floor and ceiling effects, test-retest reliability, and structural validity [16–18].

### Questionnaires

#### The university of Wisconsin running injury and recovery index (UWRI)

The UWRI evaluates the critical elements that runners use to monitor their running ability while recovering from RRI [11]. The UWRI is a 9-item questionnaire that assesses running ability following an RRI. The score ranges from 0 to 36 (a score of 36 indicates a return to preinjury running ability). The total score is calculated by summing the scores of all 9 factors. Symptom surveillance incorporates assessing and describing the psychological response (items 1, 2, 3, 4, 5 and 9). Running progression (items 6, 7 and 8) involves assessing different aspects of running through weekly volume, longest run distance, and running pace or speed [11].

#### Pain self-efficacy

Pain self-efficacy was measure with the Pain Self-efficacy Questionnaire (PSEQ), which consists of 10 items scored on a 7-point Likert scale (0–6 points) [19]. Scores range from 0 to 60, with the higher scores indicating stronger self-efficacy beliefs [20].

#### Kinesiophobia

The Tampa Scale of Kinesiophobia (TSK) measures “fear of movement” or “kinesiophobia” in the patient [21]. The total score on this scale is from 17 to 68 [22]. For example, a score of 68 showed severe fear of movement, 37 indicates there is fear of movement and where 17 means no fear [23].

#### Pain intensity

The Visual Analogue Scale (VAS) was utilized to evaluate the current pain intensity of patients. In this scale, zero indicates no pain, while ten represents the worst imaginable pain [24].

#### Translation and cross-cultural adaptation

The Persian translation and cross-cultural adaptation of the UWRI followed the Beaton multi-step process [25]. *Phase 1: Consent.* Contacted and informed original index authors of the project, and obtained consent to create a validated Persian version of the UWRI. *Phase 2: Initial/Forward translation.* Two independent Persian native speakers translate the English version of the UWRI into Persian. *Phase 3: Synthesis.* Synthesis of version 1 and version 2 to create version 3 with input from the original author and translator. *Phase 4: Back translation.* Back translation version 3 from Persian to English by two native English speakers. *Phase 5: Expert committee.* The multidisciplinary committee (composed of two translators, two research athletic trainers, an expert in research methodology/biostatistics, a research physiotherapist, and a linguist who reviews all the translations) used all versions and materials to generate an index (version 4). *Phase 6: Pre-testing.* Version 4 was pre-tested for content, wording, and understanding in a sample of patients with RRI. *Phase 7: Evaluation.* Measurement properties of UWRI-Persian (version 4) were evaluated.

#### Assessment of measurement properties

The evaluation of the measurement properties of the UWRI-Persian in a sample of patients who have RRI was guided by the COSMIN working group resources [16–18]. This study followed the Strengthening the Reporting of Observational Studies in Epidemiology (STROBE) reporting guidelines [26]. For sample size, this study followed the recommendations of the COSMIN checklist. The sample size should consist of at least 50 respondents (patients with RRI) for test-retest reliability and at least 100 respondents for studies on validity. A pre-final (pilot test) was performed with 37 patients with RRI to check whether the UWRI-Persian was understandable for them all and to collect their feedbacks.

#### Participants

Persian native speaker patients (male and female) with RRI (injury location: low back [Spinal injuries], pelvic/hip/groin, thigh, knee, shank/lower leg, ankle and foot [forefoot, midfoot, rearfoot]) participated in this study. The inclusion criteria were as follows: Persian native speaker, runners aged between 18 and 45 years old, who were willing to fill in the questionnaire [27]. Individuals were excluded if their injuries were not related to running, or if they were unable to communicate in Persian. All individuals completed the questionnaire on sports injuries and therapy. Data collection took place between May 2023 and August 2023 using flyers in physiotherapy clinics, research group networks, social media and advertisements.

### Data collection and statistical analysis

#### Reliability

Internal consistency and test–retest reliability methods were used to estimate the reliability of the UWRI-Persian. To assess test-retest reliability, the intraclass correlation coefficient (ICC) between test and retest UWRI-Persian was calculated [18]. The standard error of measurement (SEM) and minimal detectable change (MDC) were calculated according to Eq. (1) and Eq. (2), respectively. The MDC is the smallest amount of change in the scores that is not due to error in measurements [28].

Eq. 1 $$\text{S}\text{E}\text{M}=\text{S}\text{D}\, \text{p}\text{o}\text{o}\text{l}\text{e}\text{d} \times \sqrt{1-ICC}$$

Eq. 2 $${MDC}_{95\%}=\text{S}\text{E}\text{M}\times 1.96 \times \sqrt{2}$$

The test-retest reliability of the UWRI-Persian was assessed with 7-day gap between two rounds of measurements. Cronbach alpha (α) was measured to determine internal consistency. Cronbach’s α ranges from 0, meaning that the items do not correlate with each other, to 1, meaning that all items correlate perfectly with each other. An α between 0.5 and 0.6 was considered poor, between 0.6 and 0.7 was considered acceptable, between 0.7 and 0.9 was considered good, and higher than 0.9, was considered excellent [29, 30].

#### Validity

Construct validity was determined by calculating Pearson correlation coefficients at a single time point between the UWRI-Persian with PSEQ [19], TSK [22], and VAS [24]. The strength of the correlations was interpreted as weak (*r* = 0.10–0.30), moderate (*r* = 0.31–0.50) or strong (*r* = 0.51–1.00) [31]. The exploratory factor analysis was carried out using the principal component analysis method with Varimax rotation. The Kaiser-Meyer-Olkin (KMO) measure of sampling adequacy and Bartlett’s test of sphericity were performed to confirm sampling and item adequacy. The KMO measure should be greater than 0.6 [32] and the significance level for the Bartlett’s test should be less than 0.05 to ensure a satisfactory factor analysis [33].

Floor and ceiling effects were assessed. Floor and ceiling effects were considered to be present if 15% of patients reported the minimum or maximum value [30]. Descriptive statistics were calculated to determine patient characteristics. To meet the normality assumption of the data for both tests, Kolmogorov–Smirnov tests were performed. Statistical significance was set a priori at ≤ 0.05. All data were entered into SPSS (version 25.0; IBM Corp., Armonk, NY, US) for statistical analysis.

## Results

### Participants

**Fig. 1:**
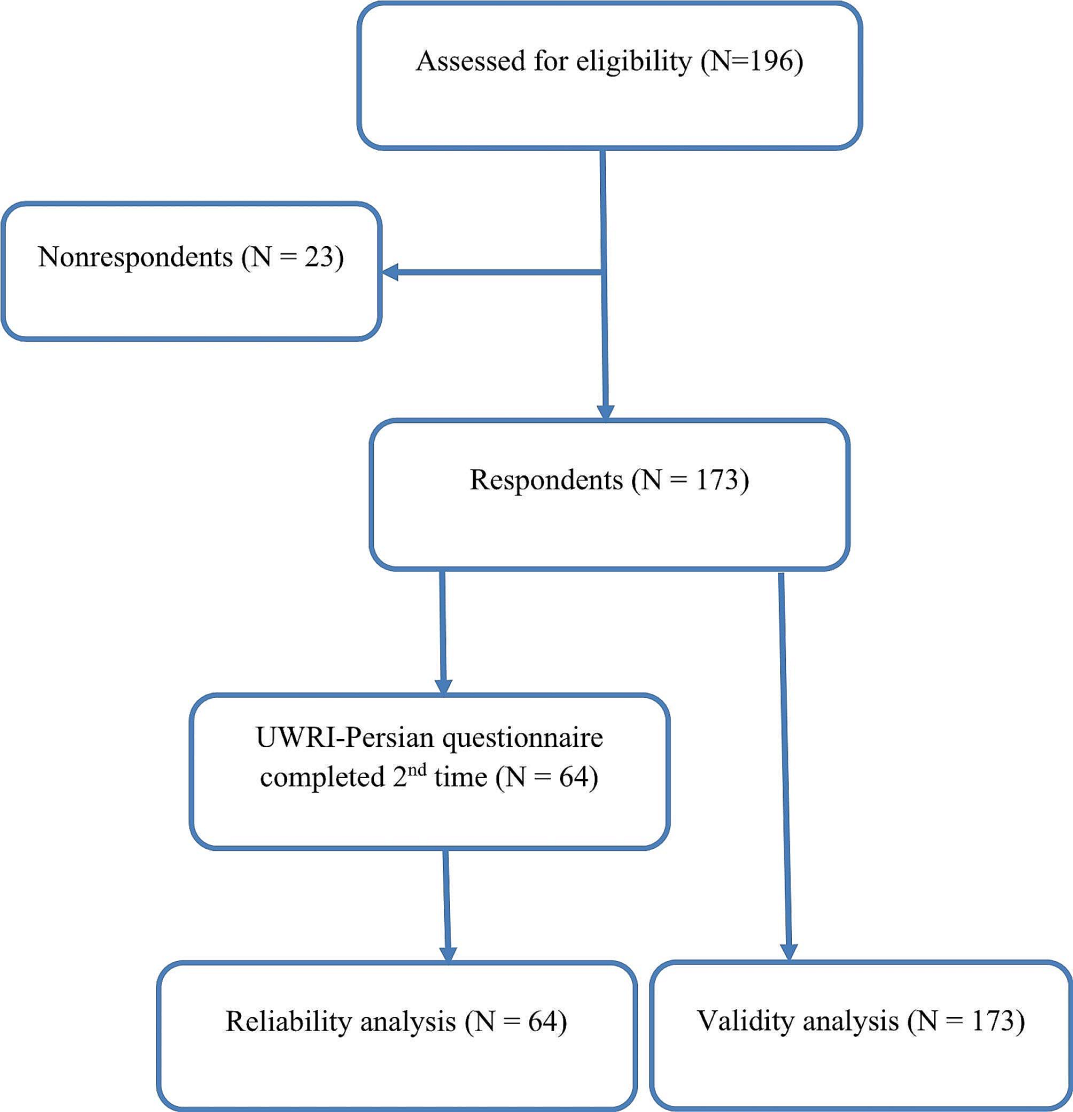
Flowchart of patients with RRI

One hundred seventy-three participants completed the UWRI-Persian, the PSEQ, TSK, and VAS at baseline. Of them, 78 (45.1%) were female and 95 (54.9%) were male with average (standard deviation) running experience of 9.8 (5.0) years. The average age of the participants was 30.1 (7.2) years. The average pain intensity was 3.9 (1.4) and the average duration of pain was 21.6 months (9.8). Sixty-four completed the UWRI-Persian questionnaire again after seven days to check reliability (Fig. 1). Table 1 shows the socio-demographic data and clinical characteristics of the participants.

**Table 1:** Demographic and clinical characteristics of the runners

Characteristic	Construct validity test (*n* = 173)	Reliability test (*n* = 64)
Age, y	30.1 ± 7.2 (18–44)	28.8 ± 6.8 (18–43)
Body mass, kg	62.3 ± 6.6 (48–77)	61.5 ± 7.2 (48–77)
Body height, cm	172.9 ± 9.9 (154–193)	171.6 ± 9.9 (156–191)
Body mass index, kg/m^2^	20.9 ± 1.4 (18.2–24.3)	20.9 ± 1.4 (18–24)
Running experience, y	9.8 ± 5.0 (3–26)	9.7 ± 5.1 (3–22)
Pain intensity (0–10)	3.9 ± 1.4 (2–6)	3.9 ± 1.4 (2–6)
Pain duration, (months)	21.6 ± 9.8 (5–40)	21.3 ± 10.1 (5–40)
UWRI-Persian (0–36)	19.9 ± 7.0 (4–36)	19.6 ± 7.0 (4–33)
Sex, n (%)		
Female	78 (45.1)	36 (56.3)
Male	95 (54.9)	28 (43.8)
Race performance, n (%)		
Ultramarathon runners	11 (4.4)	5 (7.8)
Marathon runner	28 (16.2)	11 (17.2)
Half-marathon runners	45 (26)	19 (29.7)
Long-distance runners	63 (36.4)	18 (28.1)
Middle-distance runners	26 (15)	11 (17.2)
Running level, n (%)		
Recreational runners	58 (33.5)	21 (32.8)
Novice runners	44 (25.4)	15 (23.4)
Elite runners	33 (19.1)	16 (25)
Competitive runners	38 (22)	12 (18.8)
Education level, n (%)		
High school or less	79 (45.7)	30 (46.9)
Bachelor’s degree	63 (36.4)	23 (35.9)
Master’s degree or higher	31 (17.9)	11 (17.2)
Injury location, n (%)		
Foot/forefoot/midfoot/rearfoot	20 (11.6)	7 (10.9)
Ankle	26 (15)	7 (10.9)
Shank/lower leg	32 (18.5)	12 (18.8)
Knee	40 (23.1)	17 (26.6)
Thigh	10 (5.8)	1 (1.6)
Pelvic/hip/groin	21 (12.1)	7 (10.9)
Spinal injuries/Lower back	13 (7.5)	7 (10.9)
Other	11 (6.4)	6 (9.4)

**Abbreviations**: Continuous variables were expressed as mean ± standard deviation (minimal value and maximum value) and categorical variables as number (n) and percentage (%); UWRI-Persian, Persian version of the University of Wisconsin Running Injury and Recovery Index: scores range from 0 (lower running ability) to 36 (greater running ability)

### Translation and cross-cultural adaptation

The UWRI version was translated forward and backward into Persian without any problems (Appendix 1). The translation process did not lead to disagreements between translators. The expert committee fully agreed on semantic, idiomatic, experiential, and conceptual equivalence when analysing the two translated versions. In preliminary testing, participants described the questionnaire as simple, quick and indicated that it did not contain any unclear words or awkward sentences, confirming the comprehensibility and cognitive equivalence of the translation.

### Reliability

#### Test–retest reliability

The test-retest reliability (ICC) for the UWRI-Persian total scores was 0.965 (95% CI, 0.942 to 0.979), indicating high intra-rater reliability.

#### Internal consistency

Item-to-total correlations were higher than 0.697 for all items and all correlations were positive. Cronbach’s alpha-if-item-deleted suggested that the deletion of none items did not increase α. If one item was deleted at a time, Cronbach’s and item-total correlation α ranged between 0.922 and 0.928 (Table 2).

**Table 2:** Item descriptive statistics and internal consistency of the UWRI-Persian (*n* = 173)

UWRI-Persian Items	Item mean ± SD	Item-total correlation	Scale mean if item deleted	Cronbach’s α if the item deleted
1. How does your running injury impact your ability to perform daily activities?	2.25 ± 0.92	0.704	17.69	0.927
2. How frustrated are you by your running injury?	2.24 ± 0.96	0.747	17.70	0.925
3. How much recovery have you made from your running injury?	2.24 ± 1.02	0.770	17.69	0.923
4. How much pain do you experience while running?	2.23 ± 1.01	0.777	17.71	0.923
5. How much pain do you experience during the 24 h following a run?	2.19 ± 1.01	0.759	17.75	0.924
6. How has your weekly mileage or weekly running time changed as a result of your injury?	2.23 ± 0.91	0.697	17.71	0.928
7. How has the distance of your longest weekly run changed as a result of your injury?	2.22 ± 0.96	0.734	17.72	0.925
8. How has your running pace or speed changed as a result of your injury?	2.14 ± 0.95	0.752	17.80	0.924
9. How does your injury affect your confidence to increase the duration or intensity of your running?	2.20 ± 0.97	0.792	17.74	0.922

**Abbreviations**: α, Cronbach’s alpha; SD, Standard deviation; UWRI-Persian, Persian version of the University of Wisconsin Running Injury and Recovery Index

The UWRI-Persian showed excellent internal consistency for the total score (Cronbach’s α = 0.966). Excellent internal consistency (α = 0.922) was shown for psychological response (items 1, 2, 3, 4, 5, 9) and good internal consistency (α = 0.887) for running progression (items 6,7,8). The results of measurement error reported a SEM of 1.24 points for all participants, 1.40 for psychological response and 0.90 for running progression. The MDC was 3.44, 3.87 and 2.50 for total score, psychological response and running progression, respectively (Table 3).

**Table 3:** Test-retest reliability of the UWRI-Persian subscales and total score (*n* = 64 Runners)

UWRI-Persian	First Test		Second Test		Mean difference (95% limits of agreement)	Cronbach’s α	ICC	SEM	MDC_95%_
	Mean ± SD	Min-Max	Mean ± SD	Min-Max					
**Psychological response**	13.22 ± 5.02	3 to 24	12.31 ± 4.49	3 to 23	0.906 (0.266 to 1.546)	0.922	0.914 (0.849 to 950)	1.40	3.87
**Running progression**	6.34 ± 2.41	0 to 12	6.64 ± 2.14	1 to 11	0.297 (–0.660 to 0.067)	0.887	0.884 (0.810 to 930)	0.90	2.50
**Total score**	19.56 ± 6.99	4 to 33	19 ± 6.24	6 to 32	0.563 (–0.034 to 1.159)	0.966	0.965 (0.942 to 0.979)	1.24	3.44

**Abbreviations**: α, Cronbach’s alpha; ICC, Intraclass Correlation Coefficient; MDC, Minimal detectable change with 95% confidence level; Min, Minimal value; Max, Maximum value; Psychological response, Items 1, 2, 3, 4, 5, 9; Running progression, Items 6,7,8; SD, Standard deviation; SEM, Standard Error of Measurement; UWRI-Persian, Persian version of the University of Wisconsin Running Injury and Recovery Index: scores range from 0 (lower running ability) to 36 (greater running ability)

### Validity

#### Structural and construct validity

Prior to the exploratory factorial analysis of the 9 items of the UWRI Persian, the suitability of the data for analysis was checked using Bartlett’s sphericity (*p* < 0.01) and the KMO measure of sampling adequacy (0.939). Principal component analysis revealed an underlying factor of UWRI-Persian with an explained variance of 64.92% and an eigenvalue of 5.84. The UWRI-Persian showed a moderate correlation with the PSEQ (*r* = 0.425, *p* < 0.001) and the TSK (*r* = 0.457, *p* < 0.001) and a weak correlation with the VAS (*r* = 0.187, *p* = 0.014; Table 4).

**Table 4:** Construct validity results. Correlations between UWRI-Persian, TSK, PSEQ and VAS.

Questionnaires	Mean ± SD (*n* = 173)	Correlation with UWRI-Persian (95% CI)	*p*-value
**PSEQ (0–60)**	38.27 ± 5.69	*r* = 0.425^¥^ (0.29 to 0.54)	< 0.001**
**TSK (17–68)**	30.84 ± 5.42	*r* = 0.457^¥^ (0.33 to 0.57)	< 0.001**
**VAS (0–10)**	3.87 ± 1.36	*r* = 0.187^§^ (0.04 to 0.33)	0.014*

**Abbreviations**: **, Correlation is significant at the 0.01 level; *, Correlation is significant at the 0.05 level; ¥, Moderate correlation (*r* = 0.31–0.50); §, Weak correlation (*r* = 0.10–0.30) based on the study of Cohen (1992); CI, Confidence Interval; PSEQ, Pain Self-Efficacy Questionnaire: scores range from 0 (no confidence) to 60 (high confidence); *r*, Values are Pearson’s correlation coefficient; SD, Standard deviation; TSK, Tampa scale for Kinesiophobia: scores range from 17 (no fear of movement or reinjury) to 68 (high fear of movement or reinjury); UWRI-Persian, Persian version of the University of Wisconsin Running Injury and Recovery Index; VAS, Visual analogue scale: scores range from 0 (no pain) to 10 (worst pain)

#### Floor and ceiling effects

The mean difference between the test and retest for the UWRI-Persian was 0.563 (95% limit of agreement: − 0.034 to 1.159). None of the runners reached the minimum score and only 0.58% reached the maximum score on the UWRI-Persian, implying that no floor or ceiling effects were present.

## Discussion

The aim of this study was to investigate the cross-culturally adapt the UWRI-Persian and to study its psychometric properties in patients with an RRI. The results indicate that the UWRI-Persian has good to excellent internal consistency, reliability, agreement, and construct validity. These results confirm that the UWRI-Persian is a practical tool suitable for assessing altered running ability after RRIs. This index has similar properties to the original version of UWRI [9, 34].

Psychosocial aspects such as fear of movement and self-efficacy may explain why some injured athletes take longer to rehabilitate than others. They should be evaluated in athletes who take longer than expected to complete their rehabilitation [35]. The UWRI incorporates physical and psychological factors to evaluate RRI symptoms and the impact on running. Within the running community, running distance is the principal assessment of training load [9, 34]. In this study, the validity of the different constructs of the UWRI-Persian was assessed by determining the correlation with the PSEQ, TSK, and VAS scores. Self-efficacy is recognized as one of the main psychological factors associated with return to sports [36, 37]. Woby et al. [38] found that self-efficacy beliefs mediated the impact of pain-related fear and both pain and disability outcomes. In instances where self-efficacy is low, elevated pain-related fear is likely to lead to greater pain and disability. The results of this study showed a moderate correlation between self-efficacy and the UWRI-Persian (*r* = 0.425). Alternatively, a decrease in the PSEQ score is associated with a low UWRI score and a decrease in the level of running ability after an RRI.

In individuals with chronic pain, confidence in the ability to perform specified activities correlated with the subsequent performance of those activities [39, 40]. Among runners, a common sentiment is that they fully regain their running ability once they regain the confidence to train without fear of re-injury. Maschke et al. [41] found that athletic fear-avoidance is associated with lower perceived running ability at the same time point or interval. However, one study found Kinesiophobia levels did not significantly change within anxiety levels during recovery [42]. The results obtained in this study differed from those of Madsen et al. [42] and maybe the reason for this difference is the type of study. It should not be overlooked that their study evaluated the impact of high anxiety levels on psychological state and gait performance during recovery, whereas the current study investigated the UWRI-Persian correlation with Kinesiophobia (*r* = 0.457). Also, our results showed a weak correlation between UWRI-Persian and VAS (*r* = 0. 187). This finding is consistent with a study conducted by Bunster et al. [11] who reported a weak correlation was observed between the Spanish version of UWRI and the numeric pain rating scale. In fact, this weak correlation might be attributable to the time frame that each self-reported measure uses [11]. Greater pain interference can be a product of higher fear-avoidance and would play a role in athletes avoiding rehabilitation programmes [35].

Test-retest reliability is comparable to three other translated versions and the UWRI-Persian version. The internal consistency of the UWRI-Persian is higher than that of the English [9], Spanish [11] and Turkish [12] versions, with a Cronbach’s α being 0.966 for the Persian version compared to 0.82, 0.87, and 0.84 for the other three translations, respectively. Although the internal consistency of all versions varied, all Cronbach’s α were within the recommended acceptable to excellent values [43]. The Cronbach’s α of psychological response (0.92) in the Persian version was better than in the Spanish version (0.8) [11], whereas the Cronbach’s α of running progression in the Spanish version (0.95) [11] was better than in the Persian version (0.887).

From a clinical and theoretical perspective, it has been reported that psychological factors change in patients with RRI, and these factors should be considered in rehabilitation programs [41]. The significant correlation between the UWRI-Persian and other questionnaires suggests that psychological aspects are important in determining return to running following RRI. Therefore, a valid and reliable measure of running ability after RRI may be useful in the clinical setting as a guide for the appropriate management of injured runners, although it is crucial to conduct research in this context.

### Study limitations

This study is not without limitations. The UWRI-Persian was limited to chronic or overuse running injuries, so further items should be considered before it can be used in populations with acute injury or surgery. In addition, sensitivity to alternations, and responsiveness scores were not reported in this study, which would be helpful for clinical decision making. Furthermore, conducting longitudinal studies to investigate the instrument’s sensitivity to changes over the recovery process would provide valuable insights into its responsiveness.

## Conclusion

The UWRI-Persian was successfully translated from English to Persian and demonstrated good to excellent internal consistency, validity, and reliability, with no floor or ceiling effects. This study shows that the UWRI-Persian is an instrument that can use to assess running ability after an RRI in Persian-speaking populations.

### Electronic supplementary material

Below is the link to the electronic supplementary material.

Supplementary Material 1

## Data Availability

The datasets used and/or analyzed during the current study are available from the corresponding author on reasonable request.

## Abbreviations

CI: Confidence Interval
COSMIN: Consensus Based Standards for the Selection of Health Measurement Instruments
ICC: Intraclass Correlation Coefficient
KMO: Kaiser-Meyer-Olkin
MDC: Minimal Detectable Change
PSEQ: Pain Self-Efficacy Questionnaire
RRI: Running Related Injury
SEM: Standard Error of Measurement
TSK: Tampa Scale for Kinesiophobia
UWRI-Persian: Persian Version of the University of Wisconsin Running Injury and Recovery Index
VAS: Visual Analogue Scale
