# Supplementary material for: Cross cultural adaptation, reliability and validity of the Persian version of the university of Wisconsin running injury and recovery index
Source: BMC Musculoskelet Disord. 2024 Jan 9;25:41. doi: 10.1186/s12891-024-07171-0 (PMC10775526; doi:10.1186/s12891-024-07171-0)
Supplement: Supplementary file 1 — Supplementary Material 1 [file 12891_2024_7171_MOESM1_ESM.docx]

**Appendix 1.** Persian Version of the University of Wisconsin Running Injury and Recovery Index.

| **شاخص آسیب دویدن و بهبودی دانشگاه ویسکانسین**  دستورالعمل­ها: هنگام پاسخ دادن به هر سوال، آسیب دویدن فعلی خود را در 7 روز گذشته در نظر بگیرید. گزینه مناسب را علامت بزنید(☒). | | | | | |
| --- | --- | --- | --- | --- | --- |
| 1. آسیب دویدن شما چگونه بر توانایی شما برای انجام فعالیت­های روزانه تأثیر می­گذارد؟ | □  بدون تاثیر | □  تاثیر کم | □  تاثیر متوسط | □  تاثیر زیاد | □  ناتوان نسبت به اجرا |
| 2. چقدر از آسیب دویدن خود ناامید هستید؟ | □  ناامید نیستم | □  به طور خفیف ناامید | □  نسبتاً ناامید | □  تا حد زیادی ناامید | □  به شدت ناامید |
| 3. چقدر از آسیب دویدن خود بهبود یافته­اید؟ | □  بهبودی کامل | □  بهبودی تا حد زیاد | □  بهبودی متوسط | □  حداقل بهبودی | □  بدون بهبودی |
| 4. در حین دویدن چقدر درد دارید؟ | □  بدون درد | □  حداقل درد | □  درد متوسط | □  درد زیاد | □  ناتوان در دویدن |
| 5. در طول 24 ساعت بعد از دویدن چقدر درد دارید؟ | □  بدون درد | □  حداقل درد | □  درد متوسط | □  درد زیاد | □  ناتوان در دویدن |
| 6. مسافت پیموده شده هفتگی یا زمان دویدن هفتگی شما در نتیجه آسیب شما چگونه تغییر کرده است؟ | □  یکسان یا سریعتر از قبل از آسیبم | □  به طور حداقلی کاهش یافته است | □  به طور متوسط ​​کاهش یافته است | □  کاهش زیادی داشته است | □  ناتوان در دویدن |
| 7. طولانی­ترین مسافت دویدن هفتگی شما در اثر آسیب شما چگونه تغییر کرده است؟ | □  یکسان یا سریعتر از قبل از آسیبم | □  به طور حداقلی کاهش یافته است | □  به طور متوسط ​​کاهش یافته است | □  کاهش زیادی داشته است | □  ناتوان در دویدن |
| 8. سرعت دویدن شما (زمان به مسافت یا مسافت به زمان) در نتیجه آسیب شما چگونه تغییر کرده است؟ | □  یکسان یا سریعتر از قبل از آسیبم | □  به طور حداقلی کاهش یافته است | □  به طور متوسط ​​کاهش یافته است | □  کاهش زیادی داشته است | □  ناتوان در دویدن |
| 9. آسیب شما چگونه بر اعتماد به نفس شما برای افزایش مدت یا شدت دویدن شما تأثیر می­گذارد؟ | □  اطمینان دارم که مدت یا شدت دویدن را افزایش خواهد داد | □  اگر مدت یا شدت دویدن را افزایش دهم، شاید خوب بشوم | □  فرقی نمی­کند | □  اگر مدت یا شدت دویدن را افزایش دهم، ممکن است بدتر شوم | □  من نمی­توانم مدت یا شدت دویدن را افزایش دهم |
